# Role of molecular imaging in the detection of neuroendocrine tumour

**DOI:** 10.1186/1470-7330-14-S1-O29

**Published:** 2014-10-09

**Authors:** Damian Wild

**Affiliations:** 1 Division of Nuclear Medicine, University Basel Hospital, Basel, 4052, Switzerland

## 

Neuroendocrine tumours (NETs) have distinct biological and clinical characteristics, in particular a high density of somatostatin receptors at the cell membrane [1]. It is this property that allows the use of radiolabelled somatostatin analogues for imaging of these tumours. Importantly, somatostatin receptor PET/CT imaging (e.g. ^68^Ga-DOTATOC, ^68^Ga-DOTATATE, ^68^Ga-DOTANOC) is superior to somatostatin receptor scintigraphy including SPECT/CT [2] and ^18^F-DOPA PET/CT [3] in the detection of gastroenteropancreatic neuroendocrine tumours (GEP NETs).

NETs, however, have a wide range of cellular differentiation. ^18^F-FDG PET/CT is of limited value in well-differentiated NETs but of high value in poorly differentiated NETs. Somatostatin receptor PET/CT shows contrary results [4]. As both ^18^F-FDG PET/CT and somatostatin receptor PET/CT exploit distinct tumour characteristics they are complementary for tumour staging.

Small insulinomas are difficult to detect with ^18^F-FDG PET/CT, somatostatin receptor PET/CT, ^18^F-DOPA PET/CT and morphological imaging. Targeting of Glucagon-like peptide-1 receptors using radiolabelled exendin-4 has shown to be highly effective in the detection of these tumours [5].

Clinical studies have shown higher tumour uptake of radiolabelled somatostatin receptor antagonists than somatostatin receptor agonists [6]. As a result radiolabelled somatostatin receptor antagonists may have a significant impact on imaging of NETs.
